# Single‐cell spatial transcriptomics reveals potential molecular mechanisms of *Abelmoschus manihot* (L.) medic in treating diabetic kidney disease

**DOI:** 10.1002/imt2.70099

**Published:** 2025-12-08

**Authors:** Chenhua Wu, Haitao Tang, Yihong Yu, Yuhui Song, Haitao Ge, Yiming Shen, Jie Wu, Harvest F. Gu

**Affiliations:** ^1^ Laboratory of Molecular Medicine School of Basic Medicine and Clinical Pharmacy, China Pharmaceutical University Nanjing China; ^2^ School of Life Science and Technology, China Pharmaceutical University Nanjing China; ^3^ Shandong Provincial Key Laboratory of Neuroimmune Interaction and Regulation; Department of Otorhinolaryngology, Head and Neck Surgery Yantai Yuhuangding Hospital, Qingdao University; Shandong Provincial Clinical Research Center for Otorhinolaryngologic Diseases Yantai China; ^4^ College of Pharmacy, Chemistry and Chemical Engineering, Taizhou University Taizhou China; ^5^ School of Chinese Medicine, Nanjing University of Chinese Medicine Nanjing China; ^6^ College of Pharmacy, Qilu Medical University Zibo China

## Abstract

A clinical study reported that *Abelmoschus manihot* (L.) Medic (*A. manihot*), in the form of Huangkui capsule (HKC), combined with irbesartan (IRB) is an effective therapy for patients with diabetic kidney disease (DKD). The bioactive components of HKC are total flavones extracted from *A. manihot* (TFA). To explore the pharmaceutical molecular mechanisms underlying the efficacy of *A. manihot* in the treatment of DKD, we have combined SpaTial Enhanced REsolution Omics‐sequencing (at 0.25 μm resolution) with single‐cell full‐length RNA sequencing. We employed the db/db mouse model of type 2 diabetes and DKD. These experimental methods generated the first single‐cell resolution pharmacopathological spatial atlas in kidneys of db/db mice that were treated with TFA or IRB. TFA exhibited therapeutic effects on DKD comparable to those of TFA combined with IRB. Following genome‐wide gene screening and molecular docking simulation, we have identified the key renal receptors (*Itga3*, *Itga5*, *Tgfbr1*, etc.) and regulators (*Jun*, *Junb*, *Stat1*, etc.) underlying the therapeutic action of TFA in DKD. This study provides novel insights into the pharmaceutical mechanisms of *A. manihot* in the treatment of DKD.

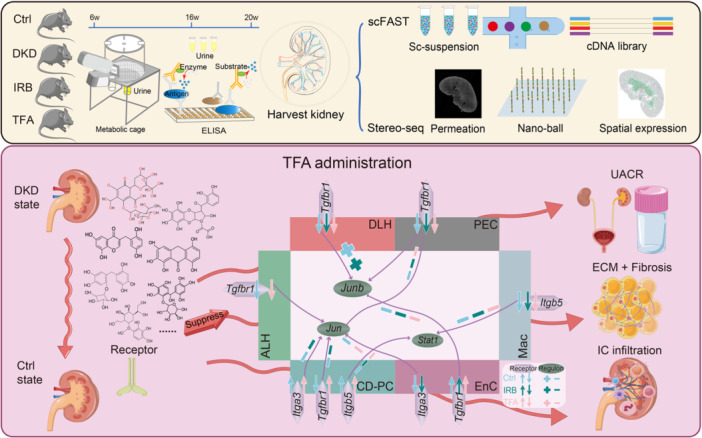

To the editor,

Diabetes, a major global public health issue primarily driven by type 2 diabetes (T2D, over 90% of cases), leads to diabetic kidney disease (DKD)—a critical complication affecting about 40% of T2D patients [1]. DKD remains one of the leading causes of end‐stage kidney disease and cardiovascular mortality, placing a heavy burden on healthcare resources, society, and family finances [2]. Currently, the clinical management of DKD requires a comprehensive strategy, including lifestyle modifications, improved control of blood pressure, key glycemic and lipid markers, and a reduction in proteinuria [3]. Traditional Chinese medicine offers unique advantages in treating DKD, particularly in holistic regulation, side effect control, symptom improvement, and delayed disease progression [4].

Huangkui capsule (HKC) is a Chinese patent medicine prepared from the ethanol extract of *Abelmoschus manihot* (L.) Medic (*A. manihot*), approved by the China National Medical Products Administration in 1999 for clinical treatment of kidney diseases, including DKD. The development trajectory of *A. manihot* is similar to that of artemisinin, both originating from ancient medical records by the Chinese physician Ge Hong, refined through modern extraction techniques, and validated via randomized controlled trials to form standardized preparations [5]. The 2015 edition of the Chinese Pharmacopoeia documents the efficacy of *A. manihot* flowers in “clearing heat, draining dampness, detoxifying, and reducing swelling.” Modern pharmacological studies, including those by our research group, have revealed that the core active components of *A. manihot* are primarily a mixture of seven flavonoids, including rutin, hyperoside, hibifolin, isoquercetin, myricetin, quercetin, and quercetin‐3‐o‐robinobioside [6, 7]. Three years ago, a multicenter randomized double‐blind parallel‐controlled clinical trial reported that HKC combined with irbesartan (IRB) effectively reduced proteinuria in Chinese T2D patients with DKD [8]. However, unlike artemisinin, which is a single compound, bioactive compounds of *A. manihot* are a total of flavonoids (TFA). Therefore, elucidating the molecular pharmacological mechanisms of *A. manihot* in treating DKD remains a challenge.

In recent years, our research group has undertaken fundamental studies of *A. manihot* for DKD treatment. We previously employed db/db mice, a widely recognized animal model for studying T2D and DKD [9], and used single‐cell RNA sequencing (scRNA‐seq) [10] to determine the ligand‐receptor‐transcription factor regulatory networks of HKC in renal cells from this model. We found that HKC modulated the downstream target genes (TG) related to immunity, fibrosis, and renal structure through regulators such as *Klf2*, *Cebpb*, and *Rel*, mediated by receptors like *Fgfr1*, *Itga1*, and *Lrp5* [11]. These findings laid the groundwork for uncovering the molecular mechanisms of *A. manihot* in DKD treatment.

## TFA POSSESSES THEORETICAL AND EXPERIMENTAL EVIDENCE COMPARABLE TO THAT OF HKC FOR THE TREATMENT OF DKD

Compared to scRNA‐seq, spatial transcriptomics (ST) enables high‐throughput reconstruction of gene expression profiles in intact tissue sections [12]. This cutting‐edge technology has been widely applied in studying the pathophysiology of complex three‐dimensional organs such as the brain, decidua, and embryos [13, 14, 15]. The kidney, as a structurally intricate organ, consists of functional units called nephrons, with distinct structural, functional, and positional differences between the medulla and cortex. The recent reports on ST analysis of DKD in humans have laid the foundation for our application of this technology to explore molecular pharmacological mechanisms implicated in DKD treatment [16, 17]. In this study, we employed 0.25 μm‐resolution SpaTial Enhanced REsolution Omics‐sequencing (stereo‐seq) and single‐cell full‐length RNA sequencing (scFAST‐seq) to construct, for the first time, a single‐cell‐resolution pathological and TFA‐treated ST atlas of kidney cells in db/db mice. The objective of this study was to improve our understanding of the molecular pharmacological mechanisms that underpin the efficacy of *A. manihot* in the treatment of DKD.

## TFA AND IRB EFFECTIVELY REDUCE IMMUNE CELLS INFILTRATION IN DKD

**Figure 1:**
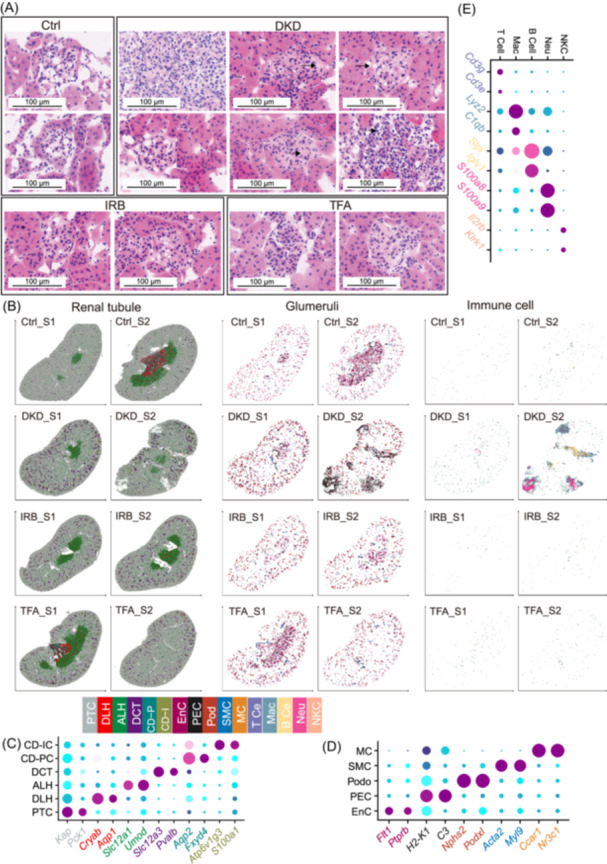
Integrated renal histopathology and spatial transcriptomics reveal the cellular landscape of diabetic kidney disease (DKD). (A) The hematoxylin and eosin (H&E) staining reveals key pathological features in DKD, including collagen deposition (Upper left, DKD panel), mesangial matrix hyperplasia, Kimmelstiel‐Wilson nodules (Lower left, DKD panel), basement membrane thickening (Upper right, DKD panel), and inflammatory cell infiltration (Lower right, DKD panel). Scale bar = 100 μm. (B) Spatial transcriptomic analysis identified 16 distinct cell types (including PTC, DLH, ALH, DCT, CD‐PC, CD‐IC, EnC, PEC, Podo, SMC, MC, T cells, Mac, B cells, Neu, and NKC) across renal tubules, glomeruli, and interstitium, visualized using their true anatomical coordinates. The *x* and *y* axes indicate only the orientation of the chip. (C–E) Cell‐type identities were confirmed using empirical marker genes. Tubular compartments contained PTC, DLH, ALH, DCT, CD‐PC, and CD‐IC (C); immune cell populations, including T cells, Mac, B cells, Neu, and NKC, localized primarily to the interstitium (D); and glomerular regions comprised EnC, PEC, Podo, SMC, and MC (E). ALH: ascending limb of Henle; CD‐IC, collecting duct intercalated cells; CD‐PC, collecting duct principal cells; Ctrl, control; DCT, distal convoluted tubule; DKD, diabetic kidney disease; DLH, descending limb of Henle; EnC, endothelial cells; IC, immune cell; IRB, irbesartan; MC, mesangial cells; PEC, parietal epithelial cells; Podo, podocytes; PTC, proximal tubular cells; scFAST, single‐cell full‐length RNA sequencing; SMC, smooth muscle cells; TFA, total flavonoids of *A. manihot* (L.); UACR, urine albumin‐to‐creatinine ratio.

Based on clinical dosage calculations [8] and our recent experimental studies [7, 11, 18], the db/db mice were randomly divided into three groups and administered daily (by gavage) with 75.5 mg/kg body weight of TFA (TFA group), 20.0 mg/kg body weight of IRB (IRB group), or an equivalent volume of purified water (DKD group). TFA was supplied by Suzhong Pharmaceutical Group Co., Ltd., while IRB was purchased from Sanofi Pharmaceutical Co., Ltd. The animal management, TFA or IRB administration, and kidney tissue acquisition procedures were conducted as previously described [7, 11, 18]. Consistent with our previous findings, treatment with TFA and IRB for 4 weeks significantly reduced urinary albumin‐to‐creatinine ratio (UACR) in DKD mice [7, 11, 18]. Hematoxylin and eosin staining revealed several pathological changes in the DKD group compared to Ctrl group (db/m mice), including glomerular sclerosis, mesangial matrix hyperplasia, collagen deposition, Kimmelstiel‐Wilson nodules, homogeneous thickening of the glomerular basement membrane, and extensive infiltration of inflammatory cells (Figure 1A). Treatment with TFA and IRB reduced the development of these pathological features in the kidneys of db/db mice, demonstrating their therapeutic effects in DKD.

After robust cell type decomposition‐based prediction and labels transformation, we retained 1,254,363 singlets from 1,468,901 cell bins and obtained cells from three sources: renal tubule (RT, 1,207,222), glomeruli (37,187), and immune cells (ICs, 9,954) (Figure 1B). We further confirmed the biological identity of these cells through the canonical cell marker (Figure 1C–E). RT included proximal tubular cells (PTC, *Kap*, *Pck1*, 1,020,959), descending loop of Henle (DLH, *Cryab*, *Aqp1*, 7,394), ascending loop of Henle (ALH, *Slc12a1*, *Umod*, 101,460), distal convoluted tubule (DCT, *Slc12a3*, *Pvalb*, 38,735), collecting duct principal cells (CD‐PC, *Aqp2*, *Fxyd4*, 19,925), and collecting duct intercalated cells (CD‐IC, *Atp6v1g3*, *S100a1*, 18,749). Glomerular cells included endothelial cells (EnC, *Flt1*, *Ptprb*, 13,359), parietal epithelial cell (PEC, *H2‐K1*, *C3*, 4971), podocytes (Podo, *Nphs2*, *Podxl*, 12,676), smooth muscle cells (SMC, *Acta2*, *Myl9*, 6,172), and mesangial cells (MC, *Ccar1*, *Nr3c1*, 9). IC included T cells (*Cd3g*, *Cd3e*, 129), macrophages (Mac, *Lyz2*, *C1qb*, 6,804), B cell (*Slpi*, *Iglv1*, 1,409), neutrophils (Neu, *S100a8*, *S100a9*, 1,569), and natural killer cells (NKC, *II2rb*, *Klrk1*, 43). The highest Pearson correlation between cell clusters from stereo‐seq and scFAST‐based cell annotation was found for corresponding cell types (Figure S1), ensuring that cell identity was sufficiently accurate to support subsequent analytical results.

## TRANSCRIPTIONAL REGULATORY PROFILES OF RENAL PARENCHYMAL CELLS IN DKD AND TFA REGULATION

**Figure 2:**
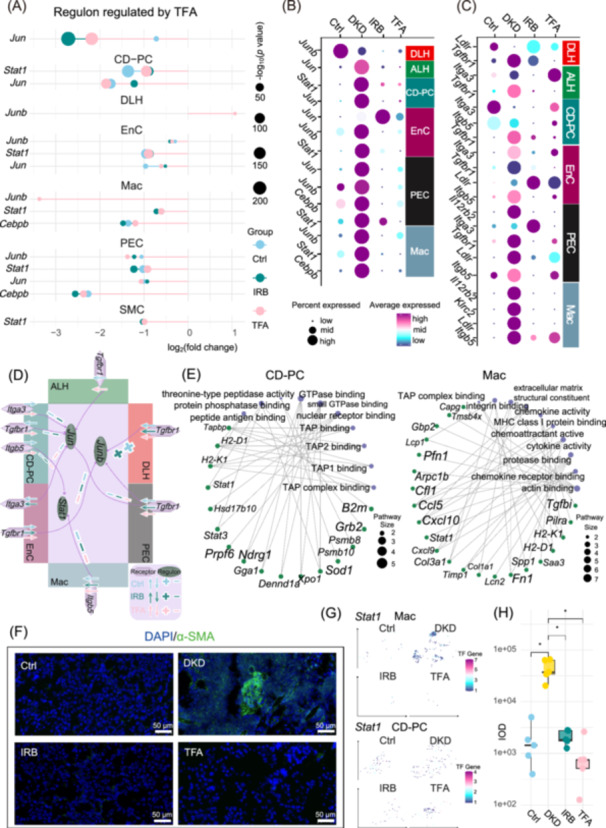
Integrative analyses of regulon activity, TF/receptor expression, spatial architecture, and functional enrichment reveal the mechanistic basis of TFA treatment in DKD. (A) Lollipop plot illustrating that a subset of the seven HKC‐regulated regulons was modulated by TFA and IRB. Bubble size denotes the *p*‐values from differential analyses relative to the DKD group. (B) Expression levels of the TF whose regulon activity differed between groups across specific cell types. Bubble size represents the proportion of expressing cells, and color indicates the mean expression. (C) Bubble plot displaying the expression of upstream receptors corresponding to the TFs in panel B across groups within the relevant cell types. (D) Schematic summarizing the up‐ and downregulation of four receptor‐regulated regulons in Ctrl, IRB, and TFA groups relative to DKD. (E) Functional enrichment of the TF Stat1 regulon, defined by the target genes displaying the highest Spearman correlations within each cell types, across GO pathways. In the cnetplot, purple nodes represent the enriched pathways, and green nodes indicate the corresponding target genes. Font size reflects gene expression magnitude. (F) Representative 40× optical images of α‐SMA immunofluorescence staining. Scale bar; 50 μm. (G) Spatial feature expression map of *Stat1* TF in CD‐PC and Mac. TF *Stat1* expression is markedly increased in DKD, and displays a spatially clustered pattern in Mac, consistently with the anatomical distribution of these cells. (H) Integrated optical density of α‐SMA (green fluorescence) across four groups (Pairwise comparisons performed using Wilcoxon tests with Bonferroni correction following a significant Kruskal–Wallis test). CD‐PC, collecting duct principal cells; DKD, diabetic kidney diseaseGO, Gene Ontology; HKC, Huangkui capsule; IRB, irbesartan; Mac, Macrophages; TF, transcription factor; TFA, total flavonoids of *A. manihot* (L.).

We then constructed the transcriptional regulatory networks and identified numerous regulons in the glomerulus (Podo, PEC, SMC, EnC), RT (DLH, ALH, CD‐PC), and IC (Mac) that exhibit significant differences between Ctrl and DKD, many of which are modulated by IRB, TFA, or both (Figure S2). We previously demonstrated that multiple regulons, mediated through specific cell–cell communication pathways, could modulate key processes in DKD, including inflammation, tubular injury, mitochondrial dynamics, and lipid metabolism [19]. In the current ST data, regulon Jun, Stat1, Junb, and Cebpb exhibited consistent trends in ALH, CD‐PC, DLH, EnC, PEC, SMC, and Mac (Figure 2A). The activity of these regulons was generally stronger in the DKD group, but suppressed in the Ctrl, IRB, and TFA groups. The corresponding transcription factors (TFs) (Figure 2B) and receptors (Figure 2C) also exhibited parallel expression patterns across the groups. For instance, the Stat1 regulon was negatively regulated by both IRB and TFA in CD‐PC, EnC, Mac, PEC, and SMC. Tracing upstream, TF *Stat1* was also identified as a differentially expressed gene in CD‐PC, EnC, PEC, and Mac. Additionally, its upstream receptor, Itgb5, a direct target of quercetin, showed the negative regulation by TFA in Mac and CD‐PC (Figure 2D). Spatial analysis revealed that TF Stat1 demonstrated a dense, high‐intensity expression pattern specifically in the CD‐PC of DKD samples. In contrast, Ctrl, IRB, and TFA groups showed only sparse, low‐level Stat1 expression. The dense expression pattern in DKD colocalized with IC distributions (Figure 2G). The target genes (TGs) of this regulon exhibited distinct functional characteristics, with the top 50 TGs most strongly co‐expressed with the TF in different cell types. In CD‐PC, the pathways related to peptide antigen binding, TAP binding, and TAP complex binding were found to be enriched in Stat1 regulon. In contrast, TGs in Mac were enriched in integrin binding, chemokine activity, cytokine activity, and other immune‐related pathways (Figure 2E). The immunofluorescence staining images showed that α‐SMA, as an important marker of renal fibrosis, had the highest signal intensity in DKD group (Figure 2F), while the fluorescence intensities in Ctrl, IRB, and TFA groups were significantly lower (Figure 2H).

The current study demonstrates that TFA improves clinical and pathological features of DKD, such as albuminuria, renal fibrosis, and extracellular matrix (ECM) deposition, by regulating IC infiltration and renal injury typically seen during DKD progression. The data indicate that TFA exhibits therapeutic efficacy comparable to both HKC and IRB in the treatment of DKD. Both TFA and IRB ameliorate the immune response and kidney fibrosis associated with DKD. Notably, the seven primary flavonoids in TFA exhibit collective inhibition of the Tgfbr1 pathway, with quercetin specifically inhibiting the Itgb5‐Stat1 pathway, thereby exerting a protective effect in DKD (Figure 2D). In this report, we provide an improved understanding of DKD pathogenesis at single‐cell level and outline several pharmaceutical mechanisms of *A. manihot* in the treatment of DKD. Chen et al. have employed scRNA‐seq in combination with the 10X visium‐based ST to analyze kidney biopsy samples from DKD patients. Their investigation uncovered the spatial correlations among vascular endothelial cells, IC, and fibrotic regions, particularly highlighting enhanced interactions between fibroblasts and three key cell types: PTC, CD‐PC, and Podo [16]. The major limitation of the 10× visium platform is its resolution at 55 μm, which often captures multiple renal cell types, such as EnC, Podo, and MC in a single spot, reducing data accuracy even with deconvolution. Abedini et al. have utilized 10× Visium‐based spatial transcriptomics at a resolution exceeding single‐cell levels to map kidney diseases (including DKD) and characterize the fibrotic microenvironment [17]. However, the method's spatial resolution was limited to 55 μm, which may restrict the ability to resolve finer cellular structures or interactions within the tissue‌.

In our previous study, we employed molecular docking and co‐expression network analysis of scRNA‐seq data to demonstrate that seven flavonoids of TFA interacted with receptors, including *Fgfr1*, *Itga1*, *Lrp5*, *Il12rb2*, *Itgb5*, *Klrc2*, *Itga3*, *Ldlr*, *Tgfbr1*, and *Nt5e* [11]. These interactions orchestrated the regulation of downstream TFs and regulons within the TG co‐expression networks, ultimately influencing key biological pathways associated with DKD, such as fibrosis, lipid metabolism, and tubular injury. In this study, we demonstrated that the TFA formulation, with its chemical composition, exhibited significant therapeutic efficacy in DKD models. During downstream regulon modulation, the TFs Jun, Junb, Stat1, and Cebpb demonstrated consistent regulatory patterns. Their upstream receptors, *Itga3*, *Itgb5*, and *Tgfbr1*, were directly or indirectly regulated by TFA. Notably, *Itgb5* expression in Mac was significantly downregulated in both the TFA and IRB treatment groups. As a pivotal receptor in cell communication via the SPP1 pathway, Itgb5 plays a critical role in mediating cellular interactions. Spp1, the ligand of the SPP1 pathway, functions as an extracellular matrix (ECM) component that actively participates in immune responses and promotes fibrotic progression. Pharmacological modulation of *Itgb5* by quercetin effectively suppressed the activity of the Stat1 regulon. Furthermore, experimental validation demonstrated that FoxO1‐mediated inhibition of Stat1 alleviated tubulointerstitial fibrosis, tubular apoptosis, and podocyte damage in diabetic mice [20]. The activation of the TGF‐β/Tgfbr1 signaling axis, accompanied by upregulation of α‐SMA and FN1, is a hallmark of renal fibrosis. TGF‐β/Tgfbr1 possesses the capacity to autonomously drive ECM and collagen deposition, making its inhibition a promising therapeutic strategy for DKD. Computational analyses further suggested plausible molecular interactions between Tgfbr1 and all seven primary flavonoids in TFA, highlighting the potential for direct targeting of this critical receptor in DKD treatment.

In conclusion, we have demonstrated that TFA treatment can ameliorate DKD in the db/db mouse model. We have identified key renal receptors and regulators that are associated with the therapeutic action of TFA in this mouse model of DKD. Our study provides a theoretical basis for the clinical application of *A. manihot*.

## AUTHOR CONTRIBUTIONS

**Chenhua Wu**: Conceptualization; software; data curation; formal analysis; investigation; methodology; writing—original draft. **Haitao Tang**: Resources; validation; writing—original draft; funding acquisition. **Yihong Yu**: Writing—original draft; methodology; data curation; visualization. **Yuhui Song**: Methodology; visualization; data curation; writing—original draft. **Haitao Ge**: Resources; validation; writing—original draft. **Yiming Shen**: Methodology; visualization; data curation; writing—original draft. **Jie Wu**: Supervision; validation; writing—review and editing. **Harvest F. Gu**: Writing—review and editing; conceptualization; project administration; funding acquisition; resources; supervision; investigation; validation; visualization. All authors have read the final manuscript and approved it for publication.

## CONFLICT OF INTEREST STATEMENT

The authors declare no conflicts of interest.

## ETHICS STATEMENT

The animal study was approved (2021‐08‐0003) by the Experimental Animal Ethical Committee of China Pharmaceutical University. The study was conducted in accordance with the local legislation and institutional requirements.

## Supporting information


**Figure S1:** The single‐cell data were clustered into 16 cell types with distinct markers, and strong correlations between scRNA‐seq and spatial transcriptomics confirmed accurate cell annotation.
**Figure S2:** The analysis compared regulon activity across groups, highlighting TFA‐regulated pathways in pink with bubble size showing *‐log_10_(*p* adj) significance.

## Data Availability

The data that support the findings of this study are openly available in the China National GeneBank Sequence Archive at https://db.cngb.org/data_resources/project/CNP0008267, reference number CNP0008267. The raw data that support the findings of this study have been deposited into the China National GeneBank Sequence Archive (CNSA) (https://db.cngb.org/data_resources/project/CNP0008267) with accession number CNP0008267. The data and scripts used are saved in https://github.com/Biomamba/iMeta2025. Supplementary materials (figures, graphical abstract, slides, videos, Chinese translated version, and update materials) may be found in the online DOI or iMeta Science http://www.imeta.science/.
