# Supplementary material for: Single‐cell spatial transcriptomics reveals potential molecular mechanisms of Abelmoschus manihot (L.) medic in treating diabetic kidney disease
Source: Imeta. 2025 Dec 8;4(6):e70099. doi: 10.1002/imt2.70099 (PMC12747534; doi:10.1002/imt2.70099)
Supplement: Supplementary file 1 — Figure S1: The single‐cell data were clustered into 16 cell types with distinct markers, and strong correlations between scRNA‐seq and spatial transcriptomics confirmed accurate cell annotation. Figure S2: The analysis compared regulon activity across groups, highlighting TFA‐regulated pathways in pink with bubble size showing *‐log10(p adj) significance. [file IMT2-4-e70099-s001.docx]

**Supplementary information to**

**Single-cell spatial transcriptomics reveals potential molecular mechanisms of *Abelmoschus manihot* (L.) medic in treating diabetic kidney disease**

**Running title***:* Single-cell spatial transcriptome profile of DKD

Chenhua Wu^1,2,3^, Haitao Tang^4*^, Yihong Yu^1,2^, Yuhui Song^1^, Haitao Ge^5^, Yiming Shen^2^, Jie Wu^2*^, Harvest F. Gu^1,6*^

^1^Laboratory of Molecular Medicine, School of Basic Medicine and Clinical Pharmacy, China Pharmaceutical University, Nanjing 210009, China

^2^School of Life Science and Technology, China Pharmaceutical University, Nanjing 211198, China

^3^Shandong Provincial Key Laboratory of Neuroimmune Interaction and Regulation; Department of Otorhinolaryngology, Head and Neck Surgery, Yantai Yuhuangding Hospital, Qingdao University; Shandong Provincial Clinical Research Center for Otorhinolaryngologic Diseases, Yantai 264000, China

^4^College of Pharmacy, Chemistry and Chemical Engineering, Taizhou University, Taizhou 225300, China

^5^School of Chinese Medicine, Nanjing University of Chinese Medicine, Nanjing 210046, China

^6^College of Pharmacy, Qilu Medical University, Zibo 255314, China

^*^Correspondence: [feng.gu@cpu.edu.cn](mailto:feng.gu@cpu.edu.cn) (Harvest F. Gu), [wujie@cpu.edu.cn](mailto:wujie@cpu.edu.cn) (Jie Wu), tanghaitao@tzu.edu.cn (Haitao Tang)

**Supplementary figures**

**
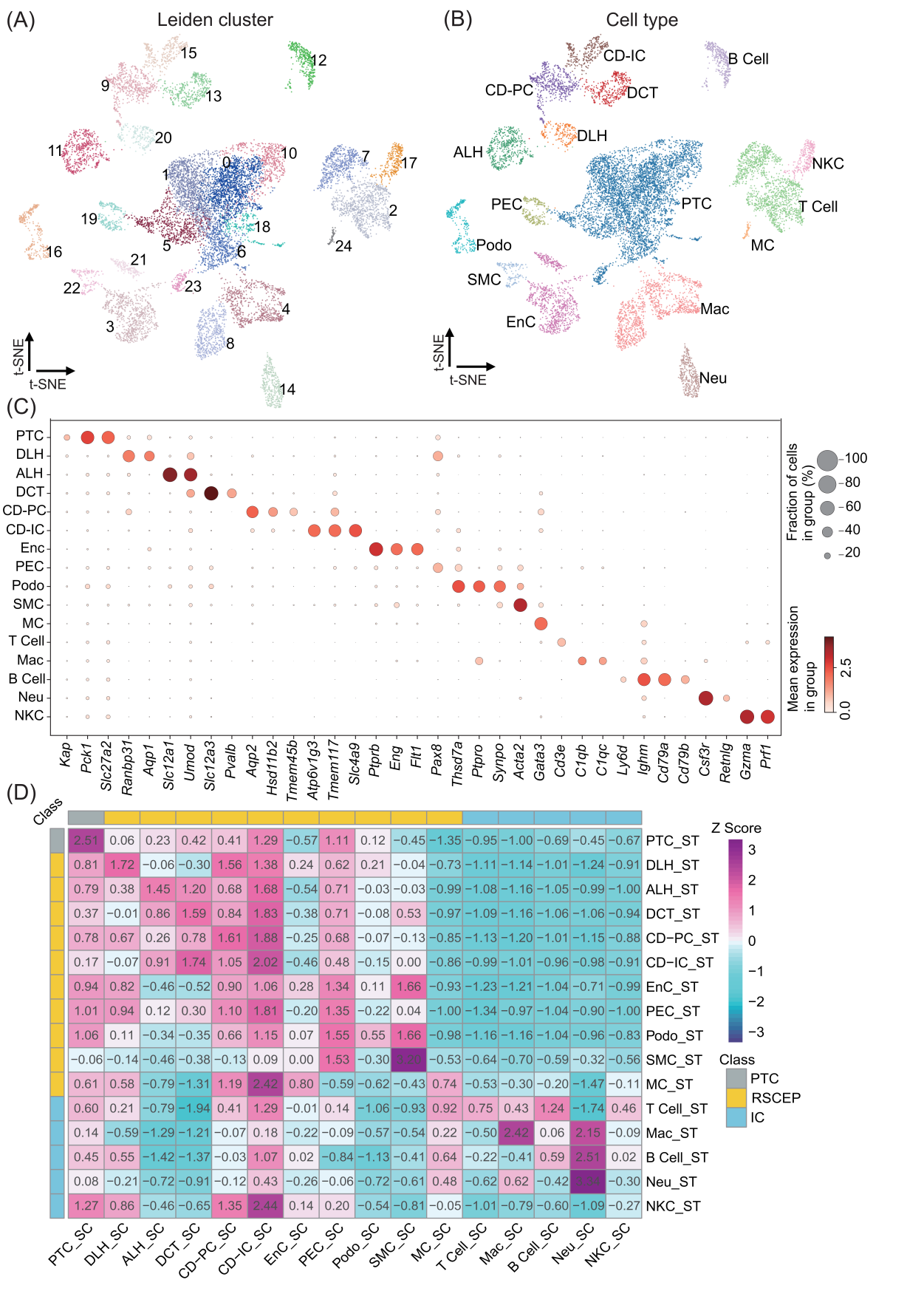
Figure S1 The single-cell data were clustered into 16 cell types with distinct markers, and strong correlations between scRNA-seq and spatial transcriptomics confirmed accurate cell annotation.** (A) After quality control to remove low-quality cells, the data were clustered into 25 clusters (0-24 clusters) using the Leiden cluster algorithm. The t-SNE plots of the clusters demonstrated substantial heterogeneity, suggesting that they likely represent distinct cell types. (B and C) These clusters were annotated with corresponding cell types (B), including PTC (*Kap*, *Pck1*, *Slc27a2*), DLH (*Ranbp3l*, *Aqp1*), ALH (*Slc12a3*, *Umod*), DCT (*Slc12a3*, *Pvalb*), CD-PC (*Aqp2*, *Hsd11b2*, *Tmem45b*), CD-IC (*Atp6v1g3*, *Tmem117*, *Slc4a9*), EnC (*Ptprb*, *Eng*, *Flt1*), PEC (*Pax8*), Podo, SMC, MC, T Cell, Mac, B Cell, Neu, and NKC. The specific markers of these renal stromal and immune cells were visualized using a bubble plot (C). (D) To validate the accuracy of cell label transfer from scFAST-seq to spatial transcriptomic (ST) data, we calculated Pearson’s correlation coefficients between the average expression matrices of matched cell populations across both two omics platforms. The row-scaled correlation matrix was visualized as a heatmap, with red hues indicating stronger inter-omics correlations. PTC: proximal tubular cells; RSCEP: renal stromal cell except PTC ; IC: immune cell; Ctrl: control ; DKD: diabetic kidney disease; IRB: irbesartan; TFA: total flavonoids of *A. manihot*; DLH: descending loop of hence; ALH: ascending loop of Henle; DCT: distal convoluted tubule; CD-PC: collecting duct principal cells; CD-IC: collecting duct intercalated cell; EnC: endothelial cells; PEC: parietal epithelial cell; Podo: podocytes; SMC: smooth muscle cell; MC: mesangial cells; Mac: macrophage; NKC: natural killer cell.

**
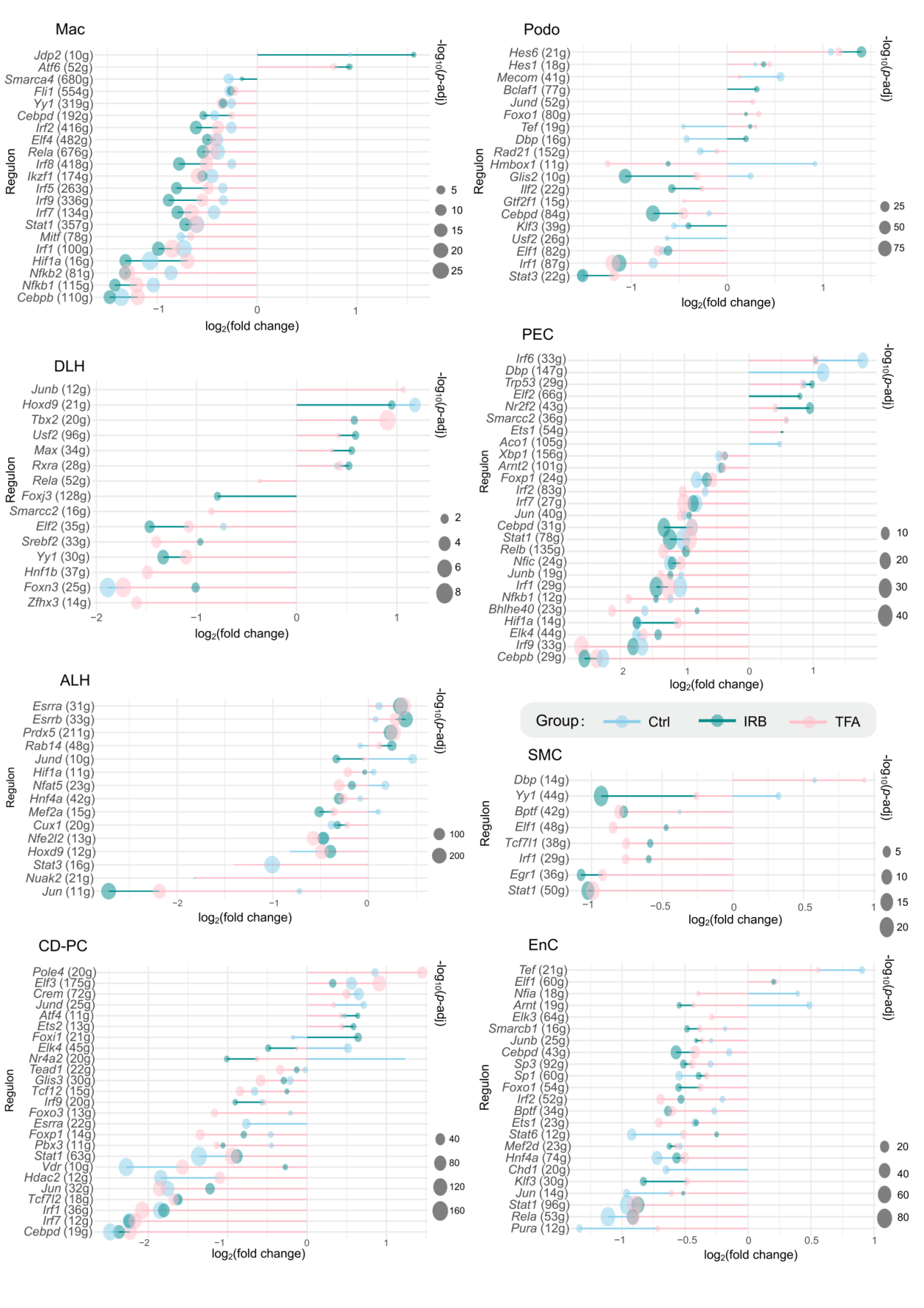
**

**Figure S2 The analysis compared regulon activity across groups, highlighting TFA-regulated pathways in pink with bubble size showing *-log_10_(*p* adj) significance.** Differential regulon activity analyses across Ctrl, IRB, TFA, and DKD groups were performed and visualized using lollipop plots. Bubble size corresponded to the *P*-value significance of regulatory divergence (*-log_10_(*p* adj)), with TFA-regulated core regulons along receptor axis pathways highlighted in signature pink for mechanistic emphasis. Ctrl: control; DKD: diabetic kidney disease; IRB: irbesartan; TFA: total flavonoids of A. Manihot; ALH: ascending loop of Henle; DCT: distal convoluted tubule; CD-PC: collecting duct principal cells; CD-IC: collecting duct intercalated cell; EnC: endothelial cells; PEC: parietal epithelial cell; Podo: podocytes; SMC: smooth muscle cell; MC: mesangial cells; Mac: macrophage; NKC: natural killer cell.
